# Exogenous Supplementation of Silicon Improved the Recovery of Hyperhydric Shoots in *Dianthus caryophyllus* L. by Stabilizing the Physiology and Protein Expression

**DOI:** 10.3389/fpls.2017.00738

**Published:** 2017-05-08

**Authors:** Prabhakaran Soundararajan, Abinaya Manivannan, Yoon S. Cho, Byoung R. Jeong

**Affiliations:** ^1^Institute of Agriculture and Life Science, Gyeongsang National UniversityJinju, South Korea; ^2^Division of Applied Life Science (BK21 Plus), Graduate School, Gyeongsang National UniversityJinju, South Korea; ^3^Research Institute of Life Science, Gyeongsang National UniversityJinju, South Korea

**Keywords:** antioxidant enzymes, proteomics, silicic acid, tissue culture, vitrification

## Abstract

Hyperhydricity is one of the major problems hindering *in vitro* propagation of *Dianthus caryophyllus* L. Silicon (Si) is a well-known beneficial element renowned for its stress amelioration properties in plants. This study has demonstrated the physiological and molecular mechanism behind the Si-mediated recovery from hyperhydricity in *D. caryophyllus* L. ‘Green Beauty’. Four weeks old hyperhydric shoots obtained from temporary immersion system were cultured on the Murashige and Skoog medium supplemented with 0 (control), 1.8 mM, or 3.6 mM of potassium silicate (K_2_SiO_3_). After 2 weeks of culture, we observed only 20% of hyperhydric shoots were recovered in control. On the other hand hyperhydricity, shoot recovery percentage in 1.8 mM and 3.6 mM of Si were 44% and 36%, respectively. Shoots in control possessed higher lipid peroxidation rate compared to the Si treatments. Similarly, damaged stomata were detected in the control, while Si treatments restored the normal stomatal development. Expressions of superoxide dismutase, guaiacol peroxidase, and catalase varied between the control and Si treatments. Furthermore, a proteomic analysis showed that as compared with the control Si up-regulated 17 and 10 protein spots in abundance at 1.8 and 3.6 mM of Si, respectively. In comparison to the 3.6 mM, 1.8 mM of Si treatment up-regulated 19 proteins and down-regulated 7 proteins. Identified proteins were categorized into six groups according to their biological roles such as ribosomal binding, oxido-reduction, hormone/cell signaling, metal/ion binding, defense, and photosynthesis. The proteomic results revealed that Si actively involved in the various metabolisms to accelerate the recovery of the shoots from hyperhydricity. Thus, the outcomes of this study can be utilized for addressing the molecular insight of hyperhydricity and its recovery mechanism by the supplementation of Si. Therefore, we conclude that active involvement of Si in the regulation and signaling process of proteins at 1.8 mM concentration could be efficient to trigger the reclamation process of hyperhydric carnation shoots.

## Introduction

Silicon (Si) is the second most abundant element in the earth crust (Epstein, 1999) and shows various beneficial effects in the plant growth and development (Ma and Yamaji, 2006). Recently, Si was listed as a “beneficial substance” or “quasi-essential” by International Plant Nutrition Institute. Effect of Si is apparently visible under several abiotic and biotic stress conditions such as drought [2.11 mM sodium silicate (Na_2_SiO_3_)] (Gong et al., 2005), salinity [1.8 mM potassium silicate (K_2_SiO_3_)] (Manivannan et al., 2016), temperature (3.6 mM K_2_SiO_3_) (Soundararajan et al., 2014), powdery mildew (1.7 mM silicic acid) (Fauteux et al., 2006), and herbivory resistance (2.7 mM NaSiO_3_⋅9H_2_O) (Keeping and Kvedaras, 2008), applied in hydroponic solution. Deposition of Si in endodermis and cell walls of root helps in selective nutrient uptake and restricts the transportation of toxic ions to the aerial parts (Yeo et al., 1999). After transpirational bypass flow, polymerized Si forms double layer with cuticle act as the physical barrier against the insects and pathogens (Ma and Yamaji, 2006). Reduction of the transpiration rate and improvement in the stomatal conductance indicate that Si strongly influence the hydraulic adjustment in plants (Ming et al., 2012). Polymerization of Si in the epidermal cells maintains the integrity of the cell membrane and water potential which prevents the electrolyte leakage (Agarie et al., 1998). The physiological improvement by Si is associated with a larger leaf area, enhanced light interception, and improved net photosynthetic assimilation (Ma and Yamaji, 2006). Addition of Si efficiently regulate the activity of the antioxidant enzymes such as superoxide dismutase (SOD), catalase (CAT), guaiacol peroxidase (GPX), and ascorbate peroxidase (APX) involved in the detoxification of excessively generated reactive oxygen species (ROS) during abnormal conditions (Zhu et al., 2004; Gong et al., 2005; Soundararajan et al., 2014, 2015). Maintenance of low molecular weight antioxidants such as ascorbate and glutathione, and proline, an important osmolyte to provide the osmotic balance (Gunes et al., 2007a). Hence, soluble Si in the xylem sap regulates various enzymatic and non-enzymatic metabolisms in the plants to maintain the proper equilibrium in the osmotic, redox, and ionic status.

Hyperhydricity is the serious problem that intrudes the *in vitro* propagation of several plants. Hyperhydric plants generally possess curled leaves with deformed, glassy, and brittle shoots containing excess amount of water. The accumulation of excessive amounts of water causes severe problems during *in vitro* propagation, organogenesis, germplasm maintenance, cryopreservation, and acclimatization (Ziv, 1991). This causes heavy loss in the medicinal, ornamental, and horticultural industries. Supplementation of Si to the tissue culture medium raise the stability of the cells, tissues, and organs (Sivanesan and Park, 2014). *In vitro* growth, biomass, and anatomy of plants were improved with the supplementation of Si under various forms such as Na_2_SiO_3_ (1.0 ppm) in *Fragaria* ×*ananassa* (Braga et al., 2009), calcium silicate (CaSiO_3_) (1000 ppm) Musa so. ‘Maca’ banana (Asmar et al., 2013), and K_2_SiO_3_ (200 ppm) for *Begonia semperflorens* and *Viola* ×*wittrockiana* (Lim et al., 2012), respectively. Oxidative stress created under the hyperhydric condition leads to the generation of ROS such as singlet oxygen ($O_{2}^{–1}$), hydrogen peroxide (H_2_O_2_), and hydroxyl radical (^_^OH) (Muneer et al., 2016). High amounts of lipid peroxidation (LPO) also cause excessive generation of ROS which damage the macromolecules including nucleic acids, proteins, and lipids (Apel and Hirt, 2004). Gunes et al. (2007a) suggested that Si efficiently reduces the LPO rate under stress conditions. During abnormal circumstances, Si assist in the maintenance of stomatal structure (Asmar et al., 2013; Manivannan et al., 2016). Ming et al. (2012) reported that Si either increase or withhold the water potential under water-deficit stress in rice seedlings. Likewise, Si supplementation involved in the maintenance of water balance during the salt stress in the tomato seedlings (Romero-Aranda et al., 2006). Enhanced activities of aquaporins present in the plasma membrane attributes to the reduced H_2_O_2_ accumulation and increased hydraulic conductance of the Si-treated tomato (Liu et al., 2015). Uptake of Si deposited mostly in the apoplast follows the water transportation (Handreck and Jones, 1968). Maintenance of water status is basis for metabolic activities in tissues (Mali and Aery, 2008). Reduction in the LPO could be caused due to the modulation in antioxidant enzymes activity and deterioration of H_2_O_2_ in the Si treatments (Gunes et al., 2007b). In cotoneaster, occurrence of hyperhydricity during the shoot multiplication was reduced by the supplementation of Si in the culture medium (Sivanesan et al., 2011). Owing to the beneficial effects of Si in several plants under various stressful environment, the current endeavor has attempted to determine the role of Si in the recovery of hyperhydricity in *Dianthus caryophyllus* L.

Carnation (*D. caryophyllus* L.) is one of the major floricultural crops and is mainly used as the cut flowers and potted plants worldwide. However, the *in vitro* propagation of carnations are highly hindered to its high susceptibility to hyperhydricity (Muneer et al., 2016). Therefore, carnations are considered as an excellent model plants to study the hyperhydricity (Olmos and Hellín, 1998). Carnation is a Si non-accumulator, since the uptake of Si studied under *in vitro* condition is merely lesser (Soundararajan et al., 2015) than the high accumulator (rice) or moderate accumulator (cucumber) (Mitani and Ma, 2005). Till now there is no molecular evidence available to predict the Si accumulation in carnation. In general, Si transporter present in the plant facilitates the massive uptake of Si in the accumulators such as rice, wheat, and sugarcane (Ma and Yamaji, 2006). However, plants with the lesser accumulation of Si could be occurred in passive mode along with the flow of water (Mitani and Ma, 2005).

Transcriptomics and proteomics analysis allows to unearth the dynamic range of changes occurred in the plant system. Transcriptomes study based on the endogenous level of H_2_O_2_ at different time interval in the hyperhydric shoots on *Allium sativum* L. revealed that genes related to the biosynthetic pathways of phytohormones such as auxin, cytokinin, and ethylene are expressed differentially during the oxidative bursting stage (Liu et al., 2017). Similarly the peach transcriptome study revealed that more than 300 transcripts were altered between the non-hyperhydric and hyperhydric leaves. Most of the transcripts modulated in peach were categorized to play vital roles in posttranscriptional process and photosynthesis, cellular elimination, cuticle development, and abiotic stress response (Bakir et al., 2016). Moreover, micro-RNA study in peach leaves suggested that around 27 miRNAs were characterized in hyperhydric leaves (Diler et al., 2016). Among them miR5021, ATP binding cassette (ABC) transporters involved in plant cuticle formation and miRnovel2, could involve in the regulation of gibberellin 2-beta-dioxygenase. As a consequence of the several disorders, hyperhydric condition induces the changes in the protein synthesis and affects the interrelated metabolic pathways (Fontes et al., 1999). Expression of Binding Protein (BiP), a member of Hsp70 protein family is higher in the hyperhydric shoots (Picoli et al., 2001). Synthesis of BiP protein is associated with the accumulation of misfolded proteins in the endoplasmic reticulum (Fontes et al., 1999). Under hyperhydric condition, antioxidant enzymes-related proteins were highly up-regulated than the normal carnation (Muneer et al., 2016). Regulation of proteins plays a key role in the developmental process of plants. Increase in the soluble protein in Si-treated cucumber plants helps to overcome the salt stress in cucumber (Zhu et al., 2004). Though, already transcriptome/proteomics studies were carried out to elucidate the influence of Si on proteins expression during the amelioration of abiotic stresses such as osmotic (Liu et al., 2015), salinity (Manivannan et al., 2016), and cadmium (Nwugo and Huerta, 2011), works on the involvement of Si on the amelioration from hyperhydricity was not conducted yet. Hence, to determine the effects of Si during the recovery of hyperhydricity the current report has focused on the physiological, biochemical, and proteomic modifications occurred during the Si-mediated recovery of hyperhydricity in carnation.

## Materials and Methods

### Plant Materials and Culture Condition

Shoots of greenhouse-grown *D. caryophyllus* L. ‘Green Beauty’ were washed with running tap water for 30 min. The excised nodal explants were soaked in Tween20 (few drops in distilled water) for 10 min and the surfactant were removed using distilled water. Inside the laminar airflow chamber explants were disinfected with 80% (v/v) ethanol for 2 min followed by 2% (v/v) sodium hypochlorite (NaOCl) containing few drops of Tween20 for 10 min. The nodal segments were thoroughly washed with double distilled water (Muneer et al., 2016). Decontaminated nodal explants were cultured on Murashige and Skoog (1962) medium consist of 3.0% sucrose (w/v) supplemented with 1.0 mg⋅L^-1^ of 6-benzyladenine (BA) and 0.5 mg⋅L^-1^ indole-3-acetic acid (IAA) in the temporary immersion system (TIS) (Plantima, A-Tech Bioscientific Company Ltd., Taipei, Taiwan) (350 mL). The immersion frequency was set to 1 min per 90 min (ABL8MEM24012, Schneider Electric, Rueil-Malmaison, France). All the cultures were maintained at 25°C and 80% relative humidity (RH) under a 16 h photoperiod with 50 μmol⋅m^-2^⋅s^-1^ photosynthetic photon flux density (PPFD) provided by cool white fluorescent lamps (40 W tubes, Philips, The Netherlands).

### Silicon Treatments

**FIGURE 1:**
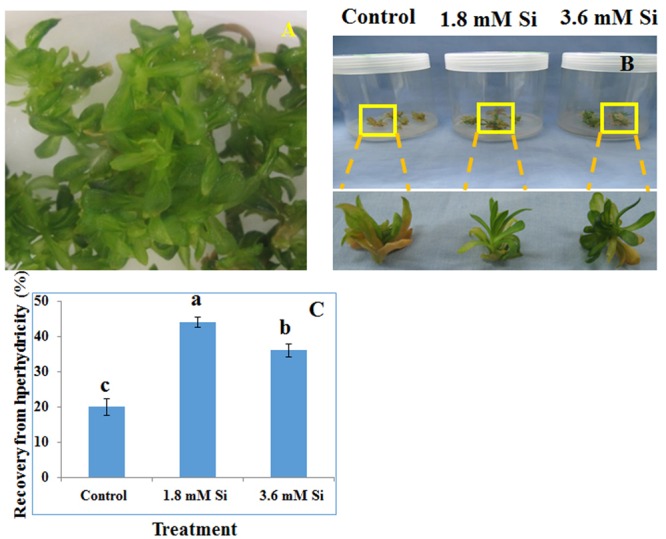
**Representative Picture of *Dianthus caryophyllus* L. ‘Green Beauty’ hyperhydric shoots (A)** and its recovery after 15 days of culture in Murashige and Skoog (MS) medium with or without Si supplementation **(B,C)**. **(A)** Induction of hyperhydric shoots in shoot multiplication medium (MS+1.0 mg⋅L^-1^ BA+0.5 mg⋅L^-1^ IAA) cultured on temporary immersion system. **(B)** Shoots cultured after 15 days on MS medium with or without Si supplementation. **(C)** Recovery percentage of hyperhydric shoots between the treatments. Different letters indicate significant difference (ANOVA, Duncan, *p* ≤ 0.05). Data are the mean ± SD from three replicates.

From our previous experiments, we have found that Si efficiently alleviate abiotic stresses (Soundararajan et al., 2014, 2015; Manivannan et al., 2016) including hyperhydricity (Sivanesan et al., 2011) at 1.8 mM and/or 3.6 mM concentration. Therefore, after 4 weeks, hyperhydric shoots (**Figure 1A**) were cultured on the solid MS medium containing 3.0% (w/v) sucrose and 0.8% agarose with 0, 1.8, and 3.6 mM potassium silicate (K_2_SiO_3_) as the source of Si. To balance the elements, the excess potassium (K) was deducted from the potassium nitrate (KNO_3_) and the loss of nitrate was balanced by the addition of nitric acid (HNO_3_). The pH of the medium were adjusted to 5.80 using 1 N NaOH or 1 N HCL before autoclave. Within 4 weeks of planting, shoots were started to dry on the MS medium devoid of Si. Therefore, all the analyses were carried out on 2 weeks old shoots.

### Scanning Electron Microscopic (SEM) Analysis of Stomata

Stomatal observations were performed using a scanning electron microscope (SEM) (JSM-6380, JEOL, Tokyo, Japan) operating at 15–25 kV. Briefly, the excised leaves were fixed in 2.5% glutaraldehyde at 4°C for overnight. Staining was done in 1.0% osmium tetroxide solution for 2 h at 4°C. After staining, the samples were dehydrated in graded series of ethanol and final wash with 80% acetone. After fixation and staining, samples were washed with 0.1 M phosphate-buffered saline (PBS, pH 7.0). Finally, the samples were dried and gold coated before the micrograph observation.

### Lipid Peroxidation and Estimation of Antioxidant Enzymes

Lipid peroxidation by measuring the content of thiobarbituric acid reactive substances (TBARS) was determined according to Zhu et al. (2004). Samples to analyze antioxidant enzymes activity were prepared according to Soundararajan et al. (2015). Nitro blue tetrazolium (NBT) inhibition method was used to determine the activity of SOD (Giannopolitis and Ries, 1977). The GPX activity was estimated based on the Shah et al. (2001). Cakmak and Marschner (1992) protocol was used to determine the activity of CAT. Enzyme activities were calculated in respective of their protein contents (Bradford, 1976). For the native-polyacrylamide gel electrophoresis (PAGE) analysis, 30 μg of protein from each treatment was mixed with a laemmli buffer (6X) at 5:1 (Laemmli, 1970). Isomers of SOD, GPX, and CAT were observed according to the Shah and Nahakpam (2012).

### Determination of Si, Macro- and Micro-Nutrients Content Using Inductively Coupled Plasma Spectrometer

To estimate the content of Si, and macro- and micro-elements, samples dried in an oven at 70°C was finely powdered using a stainless mill (Model 1093, Cytclotec, Tector, Hoganas, Sweden). Samples prepared by ashing at 525°C for 4 h (Model LV 5/11/B180, Naberthern muffle furnace, Lilienthal, Breman, Germany). The acid digestion was carried out using 20% HCl and the volume was made up to 50 mL using double distilled water. The filtered samples were analyzed using inductively coupled plasma (ICP) spectrometer (Optima 4300 DV/5300 DV, Perkin Elmer, Waltham, MA, USA).

### Proteomics

#### Protein Extraction

The finely powdered lyophilized leaf samples (100 mg) were homogenized with commercial protein extraction kit (Bio-Rad, Hercules, CA, USA) according to Manivannan et al. (2016). The content of protein was quantified by the Bradford assay (Bradford, 1976).

#### Isoelectric Focusing

For one dimensional (1D) electrophoresis, a 125 μL solubilizing buffer containing 70 μg protein was rehydrated passively for 15 h in 7 cm immobilized pH gradient (IPG) strip (pH 4–7) in the IPGbox (GE Healthcare, Little Chalfont, Buckinghamshire, UK). The focusing was carried out in the Ettan IPGphor 2 isoelectric focusing (IEF) unit (GE Healthcare, Little Chalfont, Buckinghamshire, UK) at 20°C with 50 μA per strip on the following conditions; 300 V for 0:30 (h:min) (Step and Hold), 1000 V for 0:30 (h:min) (Gradient), 5000 V for 1:30 (h:min) (Gradient), and 5000 V for 0.36 (h:min) (Step and Hold). Total time taken until final volt reaches 8.0 KVh was 3.06 (h:min).

#### Two-Dimensional Electrophoresis

Focused strips were equilibrated in a buffer [8 M urea, 2% SDS, 50 mM Tris-HCl (pH 8.8) 20% (v/v) glycerol] for the reduction with 1.0% DTT and alkylation with 2.5% iodoacetamide for 30 min at RT. Two-dimensional electrophoresis was carried out in 12.5% SDS–PAGE (PROTEAN II, Bio-Rad, Hercules, CA, USA). The proteins were visualized by silver staining and image was taken with a high resolution scanner (Epson, Long Beach, CA, USA).

#### In-Gel Digestion and MALDI-TOF/MS

By using Progenesis SameSpots 2D software (v. 4.1, Non-linear Dynamics, Newcastle, UK) differentially expressed protein spots (≥1.5-folds) among treatments were identified. The excised spots were cut into small pieces and destained with the potassium ferricyanide (30 mM) and sodium thiosulphate pentahydrate (100 mM) (30 μL) (1:1, v/v) for 30 min at RT. After one time wash with the double distilled H_2_O, gels were incubated in ammonium bicarbonate (NH_4_HCO_3_) (50 mM, v/v) and acetonitrile (ACN) for 15 min at RT, respectively. Followed by the vacuum centrifugation at 20 min, alkylation and reduction were done in 50 mM NH_4_HCO_3_ containing DTT (10 mM) for 45 min at 56°C and iodoacetamide (55 mM) for 30 min in dark at RT, respectively. After washing with 50 mM NH_4_HCO_3_, gels were shrunk by the addition of ACN for 15 min at RT and vacuum centrifuge for 20 min. Finally, peptides were digested for 30 min at 37°C using trypsin (5 ng, v/v) (Sigma–Aldrich, St Louis, MO, USA) and the overnight incubation was carried out in NH_4_HCO_3_ (25 mM, v/v) at 37°C. Aliquots were vacuum centrifuged in new microfuge tubes and the peptides were dissolved in 1–2 μL of a solvent buffer [50% ACN and 0.1% trifluoroacetic acid (TFA)].

In the matrix-assisted laser desorption/ionization time-of-flight/mass spectrometry (MALDI TOF MS)-plate (Applied Biosystems, Franklin Lakes, NJ, USA) in dark along with Angiotensin (Sigma Aldrich, St. Louis, MO, USA) standards for calibration, 1 μL of peptides were mixed with a matrix solution [10 mg⋅mL^-1^ R-cyano-4-hydroxycinnamic acid (CHCA) in 50% ACN/0.1% TFA] at 1:1 (v/v). The spots were allowed to dry after wash with the 0.01% TFA and double distilled H_2_O. Monoisotopic peaks [10 peaks per spot were selected for peptide mass fingerprint (PMF)] were obtained by the reflection of positive ion mode under the 21 kV voltage between 40 and 3000 Da mass ranges for 100 laser shots.

#### Functional Identification of Peptides

Mascot software^1^ was used to characterize the identified spots. Functions of peptides were identified under the following parameters: SwissProt; enzymes, trypsin; one missed cleavage taxonomy, Viridiplantae (Green Plants); fixed modification, carbamidomethyl cysteine; variable modification; oxidation of methionine; taxonomy, Viridiplantae; peptide mass tolerance of ±50 ppm; and protein mass of 20 KDa. The peaks from the peptide spots producing higher statistically significant (*P* < 0.05) match scores and accounting for the majority of the peaks present in the mass spectra were confirmed as the positively identified proteins. For the gene ontology (GO) annotation AgBase^2^ was used. The CIMminer online tool was used to analyze the differential expression of proteins in abundance between the treatments.

### Statistical Analysis

All experiments were set up in a completely randomized design. Each treatment was consisted of five explants with five replications per treatment. All assays were performed in three individual biological triplicates. Significant differences among treatments were determined by analysis of variance (ANOVA) followed by the Duncan multiple range test at a 5% probability level by using SAS computer package (SAS Institute Inc., Cary, NC, USA).

## Results

### Impact of Si on Hyperhydricity Recovery

**FIGURE 2:**
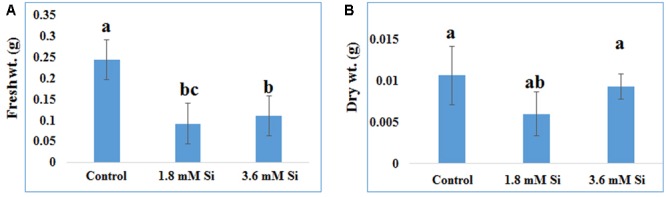
**Biomass of recovered hyperhydric shoots cultured on the MS medium with or without Si supplementation. (A)** Fresh weight and **(B)** Dry weight. Different letters indicate significant difference (ANOVA, Duncan, *p* ≤ 0.05). Data are the mean ± SD from three replicates.

Recovery percentage of the hyperhydric shoots of carnation grown under the Si non-supplemented condition (control) was lesser on comparison with the Si-supplementation treatments (**Figures 1B,C**). Nevertheless, shoots started to necrotize and dry in the medium devoid of Si from the third week of culture. However, increase in the Si concentration to 3.6 mM decreased the recovery percentage than the 1.8 mM (**Figure 1C**). Higher biomass in the control treatment represents the vitrification of shoots, i.e., excess amount of water in the tissue and the abnormal growth (**Figure 2**).

### Difference in Stomatal Structure between Control and Si Supplemented Treatments

**FIGURE 3:**
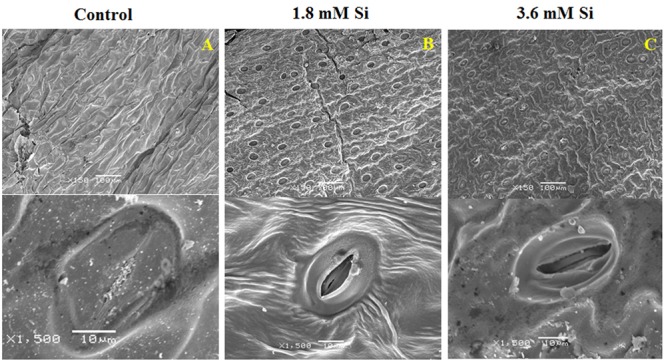
**Stomatal development in *D. caryophyllus* L. ‘Green Beauty’ after 15 days of culture in MS medium with or without Si supplementation. (A)** Control, **(B)** 1.8 mM Si, and **(C)** 3.6 mM Si.

Deformation of the stomata was observed in the control medium (**Figure 3A**). As anticipated, Si at 1.8 mM showed proper development and compactness of stomata (**Figure 3B**). Nevertheless, density and the formation of stomata were slightly affected in the medium with high concentration of Si (**Figure 3C**).

### Lipid Peroxidation and Activities of Antioxidant Enzymes

**FIGURE 4:**
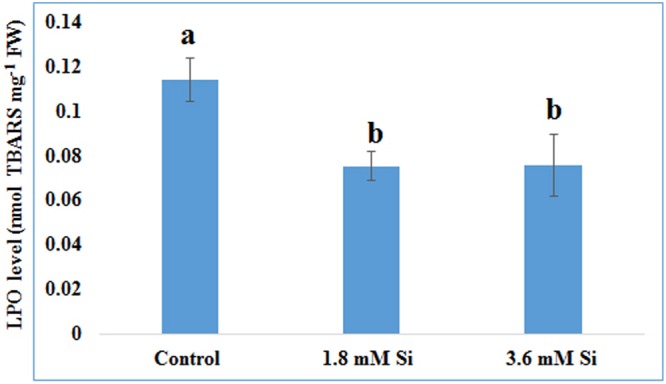
**Lipid peroxidation (LPO) of recovered hyperhydric shoots cultured on the MS medium with or without Si supplementation.** Different letters indicate significant difference (ANOVA, Duncan, *p* ≤ 0.05). Data are the mean ± SD from three replicates. TBARS, thiobarbituric acid reactive substances; FW, fresh weight.

**FIGURE 5:**
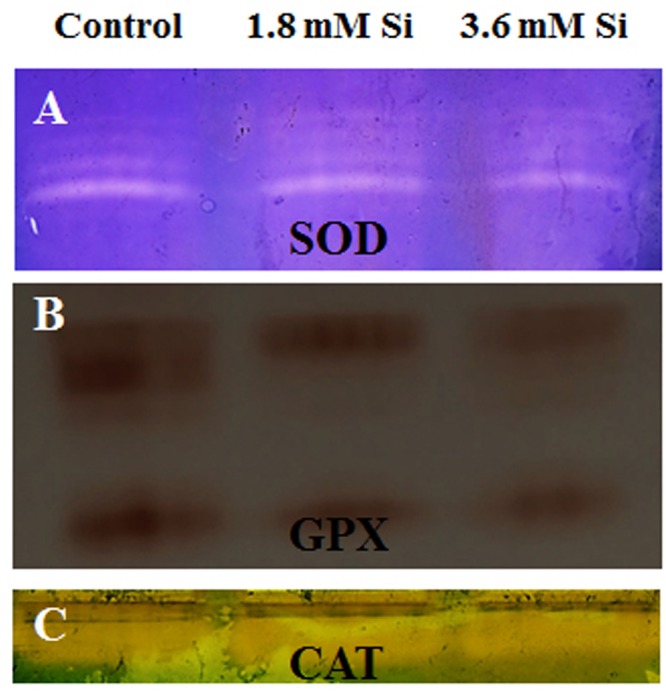
**Native-PAGE analysis of antioxidant enzymes during recovery from hyperhydricity with or without Si supplementation. (A)** Superoxide dismutase (SOD), **(B)** guaiacol peroxidase (GPX), and **(C)** catalase (CAT).

**FIGURE 6:**
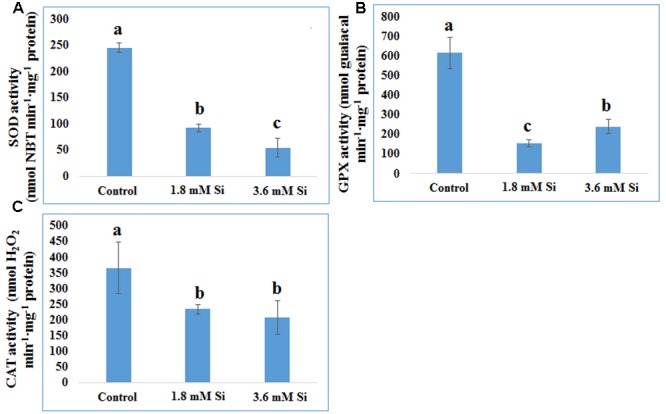
**Activity of antioxidant enzymes during recovery from hyperhydricity with or without Si supplementation. (A)** SOD, **(B)** GPX, and **(C)** CAT. Different letters indicate significant difference (ANOVA, Duncan, *p* ≤ 0.05). Data are the mean ± SD from three replicates.

Decreased LPO content (**Figure 4**) in the Si treatments denoted the reduction of oxidative stress. Expression of SOD in the 1.8 mM Si treatment was greater than the other two treatments. No significance was found between the control and 3.6 mM Si (**Figure 5A**). However, the activity of SOD was lesser in the Si treatments (**Figure 6A**). The increase in the expression and activity of GPX was observed in the control than the Si treatments (**Figures 5B, 6B**). Similarly, on comparison to the control, CAT activity decreased in the Si treatments (**Figure 6C**). However, a native-PAGE analysis of CAT showed no difference in expression among treatments (**Figure 5C**).

### Difference in Macro- and Micro- Nutrients Uptake between the Treatments

**FIGURE 7:**
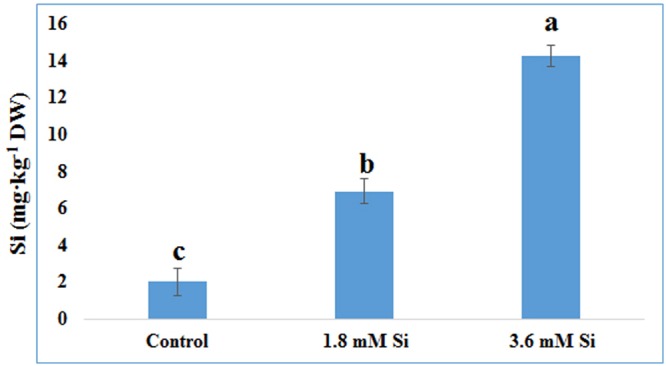
**Uptake of Si after 15 days of hyperhydric shoots cultured on the MS medium with or without Si supplementation.** Different letters indicate significant difference (ANOVA, Duncan, *p* ≤ 0.05). Data are the mean ± SD from three replicates.

The delay in the recovery process of the control is correlated with the absence of Si in the medium. The rapid recovery of hyperhydricity was directly proportional to the moderate Si content (6.9 ppm). In contrast, high level uptake of Si (14.3 ppm in 3.6 mM treatment) interrupted the recovery process from hyperhydricity (**Figure 7**). Uptake of K and sodium (Na) were increased to 511.1 and 22.1 ppm, respectively, at the 1.8 mM Si treatment. Nonetheless, availability of K and Na was decreased to 446.5 and 15.29 ppm, respectively, at 3.6 mM. The lower content of calcium (Ca) (57.1 ppm at Si 1.8 mM and 52.6 ppm at Si 3.6 mM) and magnesium (Mg) (19.7 ppm at Si 1.8 mM and 19.6 ppm at Si 3.6 mM) was observed in the Si treatments. Though the sulfur (S) uptake was lower in the Si 1.8 mM treatment than the control, phosphorous (P) availability was slightly improved in the former. In contrast, uptake of both S (32.4 ppm) and P (30.6 ppm) was affected by the presence of Si at higher concentration in the medium (**Table 1**). Among micronutrients, except Na (22.1 ppm) and Cu (0.25 ppm), not much significance was observed between control and 1.8 mM Si. Considerable improvement on the Fe uptake (4.26 ppm) was found in 3.6 mM Si treatment, meanwhile Zn content (1.11 ppm) was deeply affected (**Table 2**). Application of Si at both concentrations decreased the Mn, B, and Mo uptake than the control.

**Table 1:** Concentration of macro-nutrients after 15 days of treatment with different concentrations of Si.

Element (ppm)	Control	Si 1.8 (mM)	Si 3.6 (mM)
K	477.4 ± 7.17	511.1 ± 4.83	446.5 ± 5.00
Ca	65.5 ± 1.00	57.1 ± 0.51	52.6 ± 0.51
Mg	22.3 ± 0.27	19.7 ± 0.19	19.6 ± 0.17
S	40.7 ± 0.36	39.0 ± 0.22	32.4 ± 0.25
P	47.9 ± 0.21	48.4 ± 0.13	30.6 ± 0.36

*Data are the mean ± SD from three replicates.*

**Table 2:** Concentration of micro-nutrients after 15 days of treatment with different concentrations of Si.

Element (ppm)	Control	Si 1.8 (mM)	Si 3.6 (mM)
Na	19.40 ± 0.197	22.1 ± 0.260	15.29 ± 0.167
Cu	0.18 ± 0.002	0.25 ± 0.002	0.19 ± 0.002
Zn	1.50 ± 0.003	1.48 ± 0.007	1.11 ± 0.012
Mn	1.65 ± 0.008	1.53 ± 0.006	1.51 ± 0.015
Fe	3.77 ± 0.024	3.77 ± 0.022	4.26 ± 0.044
B	0.66 ± 0.051	0.58 ± 0.002	0.59 ± 0.008
Mo	0.07 ± 0.001	0.06 ± 0.001	0.06 ± 0.005

*Data are the mean ± SD from three replicates.*

### Functional Annotation of Differentially Accumulated Proteins in Response to Si Treatments

**FIGURE 8:**
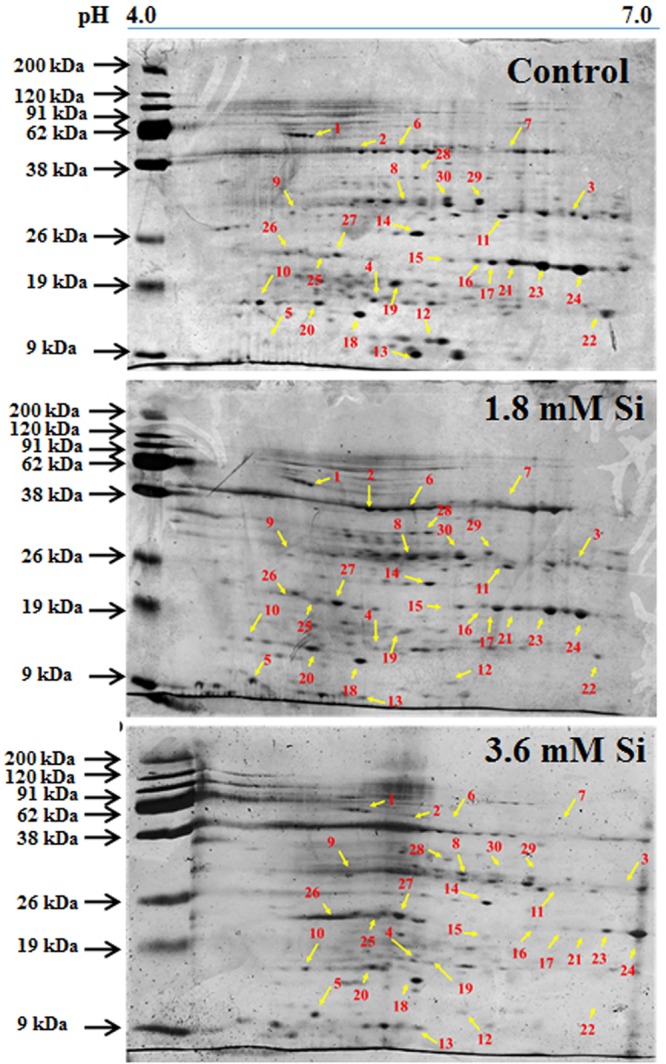
**Representative images of two-dimensional gel electrophoresis (2-DE) gel of proteins extracted after 15 days of hyperhydric shoots cultured on the MS medium with or without Si supplementation.** Differentially expressed protein spots were analyzed by the Progenesis SameSpot. Proteins with more than 1.5-fold difference among treatments identified by MALDI-TOF were listed in **Table 3**.

**FIGURE 9:**
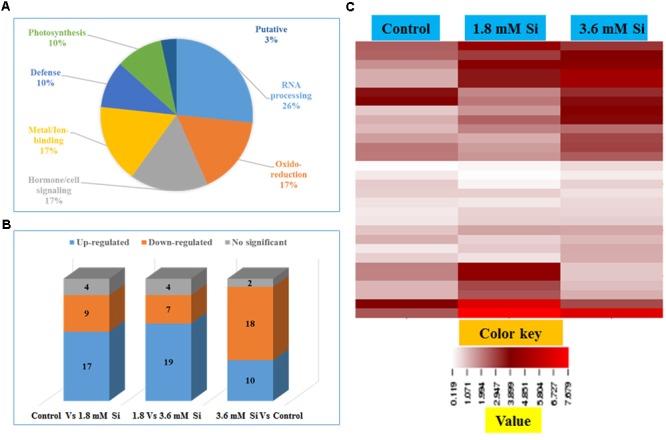
**Characterization of differentially expressed proteins in abundance between control and Si treatments. (A)** Functional classification of identified proteins by Gene Ontology analysis. **(B)** Bars with varying colors represent the up-(↑), down (↓), or non-significantly (↔) accumulated proteins in recovered hyperhydric shoots with or without Si supplementation. **(C)** Protein expression in abundance illustrated as heat map between the treatments.

**FIGURE 10:**
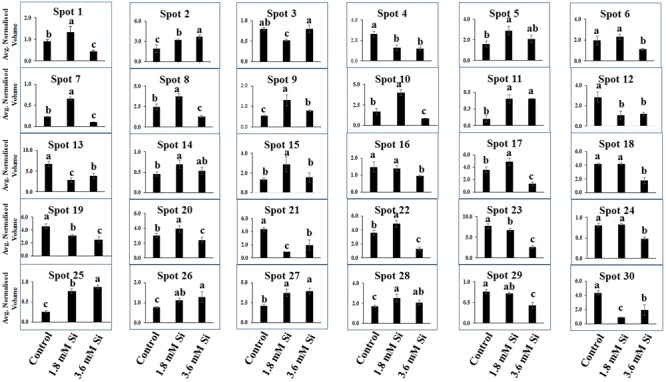
**Abundance of differentially expressed proteins of *D. caryophyllus* L. ‘Green Beauty’ during the recovery from hyperhydricity with or without Si supplementation.** Three independent experiments were performed and the mean ± SD were plotted. Different letters indicate average spot volume between the treatments are statistically different at *p* ≤ 0.05.

Totally 120 reproducible spots were detected between the treatments (**Figure 8**). Among them 30 spots significantly showed more than 1.5-fold differences between the treatments were identified using MALDI-TOF MS. Annotated proteins were classified into six groups according to their biological roles such as ribosomal binding, oxido-reduction, hormone/cell signaling, metal/ion binding, defense, and photosynthesis (**Table 3** and **Figure 9A**). Proteomic analysis showed that Si up-regulated 17 and 10 proteins in abundance in the 1.8 and 3.6 mM Si treatment, respectively. On the other hand, among the identified 30 peptides, Si treatment down-regulated 9 spots in 1.8 mM Si and 18 spots in 3.6 mM Si treatments in abundance as compared to the control. In comparison with the 3.6 mM, 19 proteins were up-regulated and 7 proteins were down-regulated in the 1.8 mM (**Figures 9B,C**). Average spot volume of the differentially expressed proteins identified between the treatments were mentioned in the **Figure 10**.

**Table 3:** Differentially expressed protein spots identified from the two-dimensional gel electrophoresis of *Dianthus caryophyllus* L. ‘Green Beauty’ shoots during recovery from hyperhydricity in Murashige and Skoog (MS) medium with or without Si supplementation.

Spot no.^a^	Accession number	Nominal mass (M_r_)^b^	Theoretical/Exp. *pI*^c^	Protein identification	Species	SC(%)^d^	Score^e^	Fold^f^
**Ribosomal binding**								
1	Q9B1K8	10809	2.8/11.10	50S ribosomal protein L23	*Lotus japonicus*	39	28	3.0
9	Q06R73	11100	2.5/11.02	30S ribosomal protein S15	*Jasminum nudiflorum*	31	22	2.9
10	Q14FC1	13613	2.05/9.35	50S ribosomal protein L14	*Populus alba*	37	29	4.6
14	Q14FC1	13613	4.1/9.35	50S ribosomal protein L14	*Populus alba*	38	32	3.3
15	B0Z5A0	10376	4.6/10.73	30S ribosomal protein S15	*Oenothera glazioviana*	34	22	2.2
12	Q5XET6	54109	6.9/9.48	RNA pseudouridine synthase 3	*Arabidopsis thaliana*	10	17	4.7
20	Q06R73	11100	3.9/11.02	30S ribosomal protein S15	*Jasminum nudiflorum*	31	23	1.6
22	Q9B1K8	10809	6.8/11.10	50S ribosomal protein L23	*Lotus japonicus*	33	32	2.7
**Oxido-reduction**								
13	O04354	14604	4.2/5.03	Cytochrome b5	*Borago officinalis*	27	29	2.4
8	Q9FLK2	7088	4.0/8.18	Probable cytochrome c oxidase subunit 5C-3	*Arabidopsis thaliana*	26	12	2.9
17	Q9FLK2	7088	5.2/8.18	Probable cytochrome c oxidase subunit 5C-3	*Arabidopsis thaliana*	33	21	2.5
29	P81247	2336	5.2/9.70	Cytochrome b-c1 complex subunit 8	*Equisetum arvense*	70	13	3.7
23	P82657	2270	6.0/8.48	Thylakoid lumenal 11 kDa	*Spinacia oleracea*	81	26	2.9
**Hormone/cell signaling**								
7	P80843	1236	5.5/9.98	68 kDa cell wall protein	*Arabidopsis thaliana*	45	15	6.3
11	P86088	1006	4.4/5.58	Alpha-amylase 2	*Capsicum chinense*	87	16	3.0
16	Q9C8Y2	20039	5.1/5.67	Protein C2-DOMAIN ABA-RELATED 2 OS	*Arabidopsis thaliana*	26	38	2.0
19	Q9ZWL6	83315	4.0/7.05	Ethylene receptor	*Passiflora edulis*	31	21	1.8
30	Q9C8Y2	20039	4.6/5.67	Protein C2-DOMAIN ABA-RELATED 2 OS	*Arabidopsis thaliana*	61	71	2.0
**Metal/Ion-binding**								
3	Q0IMG5	8379	6.5/5.62	Metallothionein-like protein 4A OS	*Oryza sativa*	58	37	2.4
4	Q8RUD6	19312	3.6/6.30	Rhodanese-like domain-containing protein 19	*Arabidopsis thaliana*	25	25	2.2
6	Q9SR25	17150	4.0/6.30	Protein AE7-like 2 OS	*Arabidopsis thaliana*	20	19	2.1
18	Q9SR25	17150	3.4/6.30	Protein AE7-like 2 OS	*Arabidopsis thaliana*	20	19	2.1
21	Q0IMG5	8379	5.6/5.62	Metallothionein-like protein 4A OS	*Oryza sativa*	47	34	3.7
**Defense**								
2	Q3ECL0	8482	3.5/9.55	Protein RALF-like 9 OS	*Arabidopsis thaliana*	18	20	1.9
5	Q8VYU8	45709	1.9/5.67	Interactor of constitutive active ROPs 5	*Arabidopsis thaliana*	11	18	1.8
27	Q4VP04	9159	3.2/8.53	Defensin-like protein 308 OS	*Arabidopsis thaliana*	45	24	1.6
**Photosynthesis**								
25	P26985	20439	7.57/0.95	Ribulose bisphosphate carboxylase small chain	*Batophora oerstedii*	53	28	3.3
26	P27063	1647	5.5/9.72	Ribulose bisphosphate carboxylase large chain	*Capsicum annuum*	62	14	2.1
28	A7M975	21497	9.27/4.10	Photosystem I assembly protein Ycf4	*Cuscuta reflexa*	28	36	1.5
**Putative**								
24	Q9LRQ1	9603	6.6/6.11	Putative BTB/POZ domain-containing protein	*Arabidopsis thaliana*	34	20	1.7

*^a^The spot nos. correspond to the numbers given in protein gel images (**Figure 8**). ^b^Theoretical molecular mass (*M*r) calculated from MASCOT Peptide Mass Fingerprint. ^c^Isoelectric point (p*I*) of spots identified from MASCOT Peptide Mass Fingerprint and protein gel images (**Figure 8**). ^d^Sequence coverage percentage. ^e^MASCOT score of protein hit. ^f^Fold change between the treatments calculated from the Progenesis SameSpots 2D software v4.1 (Non-linear Dynamics, Newcastle, UK).*

### Proteins Involved in Ribosomal Binding

Proteins responsible for the RNA processing during the translation were highly induced in abundance (spots 1, 9, 10, 14, 15, 20, and 22) in the 1.8 mM Si treatment than the control. Despite, Si at a high concentration decreased the up-regulation of proteins in spots 1, 10, and 22.

### Proteins Involved in Oxido-Reduction Process

Proteins enhanced during higher oxido-reduction period such as cytochrome b (spot 13), cytochrome b-c1 complex subunit 8 (spot 29), and thylakoid luminal 11 kDa (spot 23) were down-regulated in abundance on Si treatments. Subsequently, cytochrome c oxidase subunit 5C-3 (spots 8 and 17) was up-regulated in the 1.8 mM Si treatment than the control.

### Proteins Involved in Hormone/Cell Signaling

Abundance of alpha-amylase 2 (spot 11) increase in both 1.8 and 3.6 mM Si treatments. The down-regulation of protein domain involved in the metabolism of ABA (spots 16 and 30) was observed in the Si treatments. Decrease in abundance of ethylene receptor protein (spot 19) was observed between control and Si treatments.

### Proteins Involved in Metal/Ion Binding Process

Metallothionein-like protein 4A (spot 3) and rhodanese-like domain containing protein 19 (spot 4) were down-regulated in abundance in the Si supplemented MS medium. In contrast, no significant difference was observed between control and 1.8 mM Si treatment in protein asymmetric leaves1/2 enhancer7 (AE7)-like 2 OS protein (spots 6 and 18). Despite, the increase in Si concentration decreased the expression of protein AE7-like 2 OS (spots 6 and 18) in abundance.

### Proteins Involved in Defense Mechanism

All the identified protein spots related to defense mechanism, such as protein Rapid ALkalinization Factor (RALF)-like 9 OS (spot 2), interactor of constitutive active ROPs 5 (spot 5), and defensin-like protein 308 OS (spot 27), were up-regulated in both Si treatments than the control.

### Proteins Involved in Photosynthesis

Supplementation of Si up-regulated the ribulose bisphosphate carboxylase small chain (spot 25), ribulose bisphosphate carboxylase large chain (spot 26), and photosystem I assembly protein Ycf4 (spot 28), critical proteins required for photosynthesis. Compared with the 1.8 mM Si, expression of photosynthesis related proteins except PS I-related protein (spot 28) was slightly lesser in 3.6 mM Si treatment.

## Discussion

### Physiological Improvement Due to Si Supplementation during the Hyperhydricity Recovery Process

Although TIS was considered as a perspective technology to propagate the plants, hyperhydricity in carnation is undesirable during the shoot multiplication process (**Figure 1A**). Majority of loss to the industries occur during the acclimatization process of hyperhydric shoots (Etienne and Berthouly, 2002). Supplementation of Si improved the recovery from hyperhydricity of *D. caryophyllus* L. ‘Green Beauty’. Previously, Sivanesan et al. (2011) reported that supplementation of Si along with the shoot multiplication medium reduced the occurrence of hyperhydricity in cotoneaster. Recovery of hyperhydric shoots was higher at 1.8 mM Si treatment than the 3.6 mM. Despite, compared to the control significant improvement in the recovery was observed in high concentration Si treatment (**Figures 1B,C**). Retardation of *in vitro* growth at 3.6 mM supplementation of Si was observed in *D. caryophyllus* L. ‘Tula’ (Soundararajan et al., 2015). Decrease in growth due to the higher Si content was previously reported in several plants such as *Oryza sativa* (Agarie et al., 1992), *Vigna unguiculata* (Mali and Aery, 2008), and *D. caryophyllus* L. (Soundararajan et al., 2015). According to Yeo et al. (1999) higher deposition of Si cause the cell hardening at the earlier stage of cell elongation. Lu and Neumann (1999) reported that polymerization of Si affects the cell wall extension and lead to the cell rigidity. Deterioration in shoot growth was associated with the malformation of stomata in control (**Figure 3A**). Similar result was observed in hyperhydric shoots of *D. caryophyllus* L. ‘Dominga’ (Olmos and Hellín, 1998). Though it is not analyzed in this study, the random pattern of Si deposition in/around trichome (Montpetit et al., 2012), stomata (Zuccarini, 2008), and cell wall (Agarie et al., 1998) could resulted in the lesser recovery percentage during higher accumulation of Si (**Figure 7**). Especially, reduction in the growth and necrotic leaves were observed due to the improper regulation on the Si accumulation in *TaLsi1* transformed Arabidopsis (Montpetit et al., 2012). Therefore, delay in the recovery at the higher deposition of Si (∼14 ppm) in carnation could be due to excessive silicification.

According to Werker and Leshem (1987) the lack of recovery from hyperhydricity is linked with the abnormal stomata. Flooding of water in between the intercellular air spaces and substomatal chamber causes the stomata closure (Sibbernsen and Mott, 2010) and interrupt the mesophyll signals (Mott et al., 2008). Previously, Allen et al. (2001) mentioned that the hypertrophic state inhibit the stomatal function. Stomatal closure concurrently brings the water lodging in the apoplast. Supplemented Si could have mediated the signals from mesophyll to guard cells for releasing the blockage in the vapor-phase opening (**Figures 3B,C**). The improper gas exchange process due to deformed guard cells affects the hydrolysis or utilization of water and leads to the osmotic and oxidative stress in control. Nevertheless, shoots failed to survive in the medium devoid of Si by the fourth week of culture (Date not shown). Inadequacy of functional stomata along with hypertrophic mesophyll and protruded cell wall layer cause the death of hyperhydric plants (Fontes et al., 1999). Therefore, abnormal stomata could affect the entire physiology of the plant. Hence the facilitation of stomatal function in the Si treatment could be the prime mechanism behind the accelerated recovery from hyperhydricity in carnation.

### Regulation of Antioxidant Enzymes by Si to Overcome the Oxidative Stress

Impairment in the photosynthetic process and hypoxia condition interfere with the electron transportation during respiration and leads to the excessive accumulation of ROS such as $O_{2}^{–}$, H_2_O_2_, and ^_^OH. Oxidative stress measured with the LPO level (polyunsaturated fatty acids to form conjugated hydroperoxides) (**Figure 4**) correlated with the increased activities of antioxidant enzymes such as SOD, GPX, and CAT in control (**Figure 6**). This denotes the disproportionate presence of ROS in the carnation hyperhydric shoots cultured on Si-devoid treatment. Previously, Muneer et al. (2016) mentioned that activities of antioxidant enzymes were higher under the hyperhydric condition due to the oxidative stress increase in LPO suggested the accumulation of H_2_O_2_ and able to produce –OH via Haber–Weiss reaction (Apel and Hirt, 2004). Though antioxidant enzymes activities are substantially known for the reduction in the $O_{2}^{–}$ and H_2_O_2_ level in plants, relationship between the activity of antioxidant enzymes and tolerance was still a matter of controversy. This paradox has been raised probably due to the fact that higher activity of antioxidants correlate with both the enormous presence of ROS groups as well as detoxification of excessively accumulated free radicals (Abogadallah, 2010). For instance, SOD activity was increased under salt stress in *D. caryophyllus* L. (Soundararajan et al., 2015), decreased under prolonged exposure of drought stress in *Agrostis* spp. (DaCosta and Huang, 2007), and no effect was found in SOD under drought stress in *Poa pratensis* (Zhang and Schmidt, 1999). The variation in the expression and activity of SOD (**Figures 5A, 6A**) could be due to presence of different metal prosthetic group isoforms of SOD and their localization such as Cu/Zn-SOD (cytosol and chloroplasts), Mn-SOD (mitochondria and peroxisomes), and Fe-SOD (chloroplasts). Cavalcanti et al. (2004) reported that peroxidase enzyme was up-regulated under the salinity stress whereas CAT and SOD are either decreased or unchanged, respectively. Activities of antioxidant are mainly involved to maintain the ROS under controlled level (Abogadallah, 2010). In *A. sativum* L. higher concentration of H_2_O_2_ aggravate the hyperhydricity whereas at the lower concentration it acts as a signaling cascade to alleviate the hyperhydricity (Liu et al., 2017). Decrease in the activities of the antioxidant enzymes found on Si supplemented treatments (**Figure 6**) can be taken as controlled level of ROS maintained in the cells.

### Influence of Si on Macro- and Micro- Nutrients Content for Hyperhydricity Recovery

Presence of Si in the medium could affect both the availability and solubility of other elements present in the medium. Macronutrients are important constituents for the building blocks of plant. The improvement of K (**Table 1**) and Na (**Table 2**) uptake at 1.8 mM shows the involvement of Si in the osmoregulatory process. In addition, K also functions in the movement of guard cells for the stomata opening and closure (Siegel et al., 2009). Lesser uptake of K at 3.6 mM Si than 1.8 mM could obstruct the re-adjustment rate. Elevation of Ca content (**Table 1**) in the control is directly correlated with the stomatal deformation (**Figure 3A**) and vice versa on the Si treatments (**Figures 3B,C**). Moreover, the antagonism between the positively charged ions of K at higher level suppressed the Ca level in Si treatments (**Table 1** and **Figure 3**) could have rectified stomatal closure (Siegel et al., 2009; Franz et al., 2011). Addition of ABA elevate the cytosolic Ca^2+^-level in the guard cell induce the stomatal closure by the activation of Ca^2+^-permeable channels in the plasma membrane of guard cells (Siegel et al., 2009; Zou et al., 2010). According to the Andrews et al. (1999) under low Mg with high K, shoot growth ratio were increased prior to the root. This condition allows the accumulation of carbohydrates for the shoot development rather than the root induction and/or its development (Niu et al., 2014). Contrary to this, our previous report states that exogenous supplementation of Si to the normal shoot induced the rooting under salt stress in *in vitro* condition (Soundararajan et al., 2015). Photosystem II activity was increased under the S deficiency (Baszynski et al., 1972). Though the interaction between silicic acid and phosphate in anionic form is unlikely to occur, difference in the P level between the two Si treatments probably caused due to change in rate of P-translocation (Ma et al., 2001).

Micronutrients are indispensable components of enzymes and also act as secondary messengers. Recovery from the hyperhydricity mediated by Si are both from the concomitant improvement and restriction in the uptake of micro-nutrients (**Table 2**). Franz et al. (2011) reported that the highest Cu treatment of Zinnia with Si supplementation plants are grown healthier. Silicate ameliorated the excessively presented Zn in the cytoplasm by co-transportation into the extracellular compartments (Neumann and zur Nieden, 2001). Si application increased binding of Mn to cell wall and enhanced distribution prevents Mn to reach its toxic level in *V. unguiculata* (Iwasaki et al., 2002). Modification of cation binding properties of the cell wall in Si treatments lead to the Mn reduction (Rogalla and Römheld, 2002). Boron-silicate (B-Si) complex formation in the soil decreased B uptake in *Spinacia oleracea* L. (Gunes et al., 2007a). Since the pros and cons of organic elements are determined by either lower or excess availability of one or more ions, the varying results obtained in the macro- and micro- element contents could be due to the hyperhydric nature of shoots as well as Si treatments.

### Possible Proteomic Regulatory Network of Si for the Adaptation of Hyperhydric Shoots

To plot the critical view point of hyperhydric shoots adaptation in the Si supplemental medium, proteomics analysis was carried out. Functionally categorized peptides details were shown in **Table 3**. Abundance of proteins involved in various metabolisms and their network signals the response of plants to the external stimuli. More than one spots matching to the same protein/peptide could be due to isoforms, subunits, maturation, degradation, and/or post-translational modification of proteins (Nwugo and Huerta, 2011).

Ribosomal binding proteins are depressed under Si devoid condition. Ribosomal proteins plays an important role in the flow of biological information (Bachellerie et al., 2000). Cellular component of the identified 50S and 30S ribosomal proteins (**Table 3**) is chloroplast. Maximum number of proteins in the chloroplast are involved in the photosynthetic electron-transport complexes and ATPase/NADPH complexes. Reduction in the accumulation of chloroplastic ribosomal proteins are directly related with the lesser turnover or abundance of ribulose 1,5-bisphosphate carboxylase (RuBisCo) (spots 25 and 26), which interrupt the physiological process (Pospisilova et al., 1992). Pseudouridine (5-ribosyluracil) is the modified RNA, i.e., polynucleotide chain found in *anti*-configuration. It function as the conformational switch during the low energy requirement for the *syn*/*anti* transition in RNA (Charette and Gray, 2000). The abundance of ribosomal proteins could bring the remarkable changes in the transition of hyperhydric to normal shoots. However, takeoff of this process is delayed during the initial period of 3.6 mM Si treatment (**Figure 10**).

Cytochrome b was proposed as the main producer of $O_{2}^{–}$ radicals in the peroxisomes (López-Huertas et al., 2000). Peroxisomal membrane polypeptides uses NADH as an electron donor for $O_{2}^{–}$ generation and able to reduce the cytochrome c (del Río et al., 2002). Upregulation of thylakoid protein indicates the higher phosphorylation of light harvest complex II (Jensen et al., 2007) and excessive presence of hydroperoxides (Peltier et al., 2002). This occurred during the excited state transitions induced by the binding of reduced plastoquinone products with cytochrome b_6_/f (López-Huertas et al., 2000). Therefore, decreased abundance of cytochrome b complex (spots 13 and 29) and thylakoid protein (spot 29) up-regulated the cytochrome c (spots 8 and 17) (**Table 3** and **Figure 10**) shows the maintenance of equilibrium on ROS generation in Si treatments.

The increase in the abundance of 68 kDa cell wall protein in the 1.8 mM Si treatment depict the accustomed progress in cell development (Irshad et al., 2008) of Si treatments (**Table 3** and **Figure 10**). Meanwhile, lesser expression of cell wall protein acquainted the restriction in the plasticity and cell elongation in control and 3.6 mM Si treatment. Synergistic repression of ABA signaled by alpha-amylase 2 promoter was found in rice (Xie et al., 2006). Similarly, higher abundance of alpha-amylase (spot 11) and the restraint of ABA-related proteins identified in this study. Augmentation of ABA maximizes the ability of Ca^2+^ to down-regulate the inward K^+^ by activating the S-type anion channels (Siegel et al., 2009). Allen et al. (2001) defined that Ca^2+^ program the long-term inhibition of stomata opening. Regulation of stomatal movement by the inward K^+^ current could prevent the stomata closure in the Si treatment (**Figures 3B,C**) was correlated with the inhibition of ABA and Ca^2+^-mediated signaling (**Figure 9C**). Attenuation in the stomatal and excessive wateriness of shoots on control was also corresponds with the higher abundance of ethylene receptor. Increased ROS production activates the ACC synthase, a precursor for ethylene (Kim et al., 2008). Abscission of leaves and shoot death observed in the control can be correlated with the ethylene receptor (spot 19) abundance in the control. Higher H_2_O_2_ production by the overexpression of Chloroplast Cu/ZnSOD could trigger the ethylene production (Kim et al., 2008). Nevertheless, proper regulation of plant hormone-, ion-, and ROS-signaling mediated by Si could attenuate the abscission rate.

Increase in the metallothionein-like protein in control and 3.6 mM Si treatment indicates the requirement of metal homeostasis. Higher abundance of metallothionein instigate the requirement of metalloenzymes to scavenge the excessively accumulated ROS groups (Zhou et al., 2006). Previously, Okumura et al. (1991) reported that Cu treatment decreased the expression of metallothionein in mRNA level in barely. Similarly, higher Cu content (**Table 2**) observed in the 1.8 mM of Si showed lesser abundance of metallothionein-like protein (spot 3) in carnation. Protein AE7 (spots 6 and 18) participates in the cytosolic Fe-S cluster assembly for the maintenance of genome integrity (Luo et al., 2012). No significance between the control and Si 1.8 mM treatment in Fe and S is correlated with the expression of protein AE7. However, this process could be insubstantial (**Table 2**) in the higher Si supplemented treatment (**Figure 7**). Meanwhile, the higher abundance of rhodanese-like domain containing protein 19 (spot 4) could lead to the formation of sulfite and thiocyanate (Papenbrock et al., 2011). Notably, rhodanese protein upregulation in the control (**Table 3** and **Figure 10**) specifies the oxidization of sulfite in non-enzymatic manner by H_2_O_2_ (Hänsch et al., 2007). Receptor mediated polypeptide signals (RALF protein, spot 2) regulate the defensive and developmental process on Si treated shoots (Pearce et al., 2001). Similarly, increased in the abundance of interactor of constitutive active ROPs 5 instigate overall physiological development as it is involved in the diverse signaling cascades includes growth and polarity establishment of cell, morphogenesis, cytoskeleton organization, hormone signaling, and other vital cellular processes (Zheng and Yang, 2000). Induced expression of defense-related proteins is an integral part enabling the plant to cope-up instantaneously against the stress.

Reduction in the photosynthesis-related protein in control signifies the poor photosynthetic capacity. RuBisCo plays an important role in the assimilation of CO_2_ and conversion of starch (Raines, 2011). Similarly, photosystem complex are vital for light harvesting and the photoprotection (Kozaki and Takeba, 1996). Pospisilova et al. (1992) reported that abnormal development of photosynthetic apparatus is the main reason for the low photosynthetic efficiency. In the Si treatment significant correlation was observed between the improvement in the expression of photosynthesis related proteins such as RuBisCo large (spot 25) and small (spot 26) subunit and photosystem I assembly protein Ycf4 (spot 28) and stomatal developmental (**Figures 3B,C**) (Raines, 2011). Decrease in the respiratory activity in hyperhydric shoots could reflect the lesser reduced and oxidized pyridine nucleotides. Production of NADPH and NADP^+^ might affected in the control and this process was precisely retrieved in 1.8 mM Si treatment. Therefore, the dignified improvement on the convalescence of hyperhydric shoots in Si treatments depends on the enhanced expression of photosynthetic proteins in abundance and movement of stomata.

## Conclusions

The current endeavor demonstrate various factors related to the amelioration of hyperhydricity with the supplementation of Si in *D. caryophyllus* L. ‘Green Beauty’. Facilitation of stomatal opening assisted by the Si supplementation could be the decisive factor for the upregulation of photosynthesis-related proteins. Accelerated re-establishment from the hyperhydricity and progressive physiological development on the Si treatments in *D. caryophyllus* L. was aid by the regulation of nutrient uptake. Minimal LPO content and normal activities of antioxidant enzymes illustrate the equilibrium in redox homeostatic process. Lack of cell elongation and plasticity along with the reduction in the uptake of K under higher concentration of Si could contempt the rapid recovery from hyperhydricity. Active involvement of Si in the regulation and signaling process of proteins in different metabolisms prevents the aggravation of hyperhydricity. From this study, it can be concluded that Si at 1.8 mM could be the optimal concentration to trigger the reclamation process and favorable conditions for the survival of hyperhydric carnation shoots.

## Author Contributions

PS, AM, and BRJ conceived and designed the experiments; PS and AM conducted the experiment, collected, and analyzed the data; PS wrote the draft of the manuscript; YSC assisted in conducting the experiment, sample collection, and all the analysis; AM and BRJ proofread and finalized the manuscript.

## Conflict of Interest Statement

The authors declare that the research was conducted in the absence of any commercial or financial relationships that could be construed as a potential conflict of interest.

## Footnotes

1 www.matrixscience.com

2 http://www.agbase.msstate.edu/
